# Effects of resveratrol-loaded dendrimer nanomedicine on hepatocellular carcinoma cells

**DOI:** 10.3389/fimmu.2024.1500998

**Published:** 2024-11-14

**Authors:** Jiao Qu, Yueqin Zhang, Cong Song, Yue Wang

**Affiliations:** ^1^ Department of Radiology, Songjiang Hospital Affiliated to Shanghai Jiao Tong University School of Medicine, Shanghai, China; ^2^ Medical Science and Technology Innovation Center, Shandong First Medical University, Jinan, Shandong, China

**Keywords:** dendrimers, resveratrol, hepatocellular carcinoma cells, cancer cells, nanomedicine

## Abstract

Resveratrol (Res), a Chinese herbal extract, has demonstrated a remarkable and distinct antitumor effect, characterized by prolonged efficacy and minimal adverse reactions. However, the bioavailability of Res in animals is hindered by limited absorption rates. Therefore, it is crucial to enhance the tumor-targeting ability of resveratrol to optimize cancer treatment outcomes by improving its bioavailability. Herein, we attempt to employ a functionalized nanoparticle drug carrier system based on polyamine-amine (PAMAM) dendrimers for targeted delivery of resveratrol in hepatocellular carcinoma cancer treatment. Initially, galactose-modified fifth-generation (G5) PAMAM dendrimers (G5-Gal) were synthesized through coupling reactions, followed by the synthesis of glycosylated dendrimers incorporating resveratrol (G5(Res)-Gal) *via* physical encapsulation. The G5-Gal or G5(Res)-Gal complexes were characterized using ^1^H NMR spectroscopy, zeta and size analysis, and UV spectrophotometry. Additionally, Hepa1-6 mouse hepatoma cells were utilized as model cells to assess the targeting capability of G5-Gal toward hepatoma cells using flow cytometry. Finally, CCK-8 assay was employed to evaluate the impact of G5(Res)-Gal on normal liver cells as well as its cytotoxicity against different types of hepatoma cells. Furthermore, cell apoptosis experiments were conducted to further evaluate the effects of G5(Res)-Gal on Hepa1-6 cells. The aim of this project is to establish a solid theoretical framework and provide technical expertise to optimize the application of resveratrol and advance its delivery system.

## 1 Introduction

With the passage of time, cancer has emerged as the foremost peril to human health and normal life. Among these malignancies, hepatocellular carcinoma stands as the second leading cause of cancer-related mortality worldwide, with its incidence and fatality rates escalating annually (1, 2). The pathogenesis of hepatocellular carcinoma primarily stems from aberrant hepatocyte hyperplasia; if left uncontrolled, this abnormal proliferation culminates in intracellular mutations that give rise to hepatic neoplasms. The pathogenesis of hepatocellular carcinoma primarily arises from aberrant hepatocyte hyperplasia; if left uncontrolled, this abnormal proliferation ultimately leads to intracellular mutations that give rise to hepatic neoplasms (3). Moreover, various chemicals prevalent in modern lifestyles such as plastics, preservatives, and hormones are also deemed significant etiological agents for hepatocellular carcinoma. According to the Global Cancer Statistics 2020 data analysis report, in most regions globally, male individuals exhibit two to three times higher incidence and mortality rates compared with their female counterparts. The fact that hepatocellular carcinoma has risen from being ranked third highest in terms of mortality rate among all cancers in 2018 to becoming the second highest by 2020 is particularly concerning (1). Consequently, the development of effective treatment strategies aimed at reducing mortality in hepatocellular carcinoma patients remains a paramount objective for biomedical researchers both within China and globally.

The current primary clinical approaches for the treatment of hepatocellular carcinoma include surgical resection, interventional therapy, radiotherapy, and chemotherapy. Among them, the administration of cytotoxic drugs, known as chemotherapy, involves the use of chemicals throughout or in specific areas of the body to inhibit tumor growth or eradicate tumors (4). Although the main objective of chemotherapy is to reduce tumor burden, this treatment method exhibits limited efficacy against cancer cells due to its inability to specifically target them. Consequently, chemotherapy always results in substantial damage to normal tissues and cells while killing cancer cells. Patients often experience adverse effects such as nausea, vomiting, and hair loss. Moreover, the administration of chemotherapy is constrained in its clinical application for hepatocellular carcinoma due to the presence of various immediate signs and long-term indications of toxicity, which impose significant limitations on its use. Additionally, the use of chemotherapy is further constrained in its clinical application for hepatocellular carcinoma due to the presence of various immediate signs and long-term indications of toxicity, which impose significant limitations on its use. Therefore, the current challenge lies in enhancing the anticancer efficiency of drugs while minimizing toxic side effects on normal tissues.

A considerable number of studies have demonstrated that certain traditional Chinese medicines exhibit superior and distinctive antitumor effects, characterized by long-lasting efficacy and minimal side effects. However, the complexity of their chemical composition poses a drawback as it contains both a variety of effective and ineffective ingredients, along with toxic components. Therefore, extracting the active monomers from Chinese medicine can maximize its therapeutic potential. For instance, resveratrol (Res), also known as 3,4,5-trihydroxystilbene, is a natural polyphenol found abundantly in various plants such as peanuts, blueberries, cranberries, beans, rhubarb grapes, and eucalyptus trees, with grapes having the highest content (5). Resveratrol was initially isolated from veratrum root in 1940 and later extracted from the root of *Polygonum cuspidatum* plant in 1963 (5, 6). Resveratrol exists in two geometric isomers—its cis-isomer being unstable whereas its trans-isomer exhibits higher stability and biological activity. The chemically synthesized pure form of resveratrol is currently available for biomedical applications. It exhibits a wide range of pharmacological activities, including immune enhancement, deceleration of the aging process, platelet depolymerization, antioxidant properties, anti-inflammatory action, vasodilation effect, and anticancer properties (5, 6). Nevertheless, resveratrol’s strong physiological activity is hindered by drawbacks like rapid metabolism within the body poor oral absorption capacity and limited water solubility, which restricts its clinical application and development (7, 8). Hence, it becomes crucial to establish an efficient drug delivery system for resveratrol to enhance targeting capabilities and bioavailability.

The field of nanomedicine provides innovative nanotechnology approaches for the diagnosis, prevention, treatment, and eradication of life-threatening diseases. Various nanoplatforms including liposomes (9), dendrimers (10, 11), polymer micelles (12, 13), nanogels (14, 15), and carbon nanotubes (16–18) have been extensively utilized in the field of cancer diagnosis and treatment. Dendrimers are highly branched, three-dimensional, monodisperse macromolecules that iteratively extend from the core (19). The main categories of dendrimers include polyamide-amine (PAMAM), polypropylene imine (PPI), polyester, and amino acid dendrimers (20). Among these types, the PAMAM dendrimer has been widely studied since its first synthesis in 1985 by American scholar Tomalia D.A. (21, 22). The PAMAM dendrimer typically consists of a centrally located core, surrounded by a nucleus, shell, and branches, with the core serving as the focal point for its branches. Hydrophilic layers can be constructed with dendrimer shells to efficiently encapsulate small RNAs and drugs (23). Furthermore, amino-terminated PAMAM dendrimers readily bind to related ligands through chemical reactions enabling targeted drug delivery to cancerous tissues/cells while minimizing toxic side effects on normal tissues/cells (24). Consequently, PAMAM dendrimers have found extensive applications in biomedicine particularly in targeted drug delivery (25).

**Figure 1:**
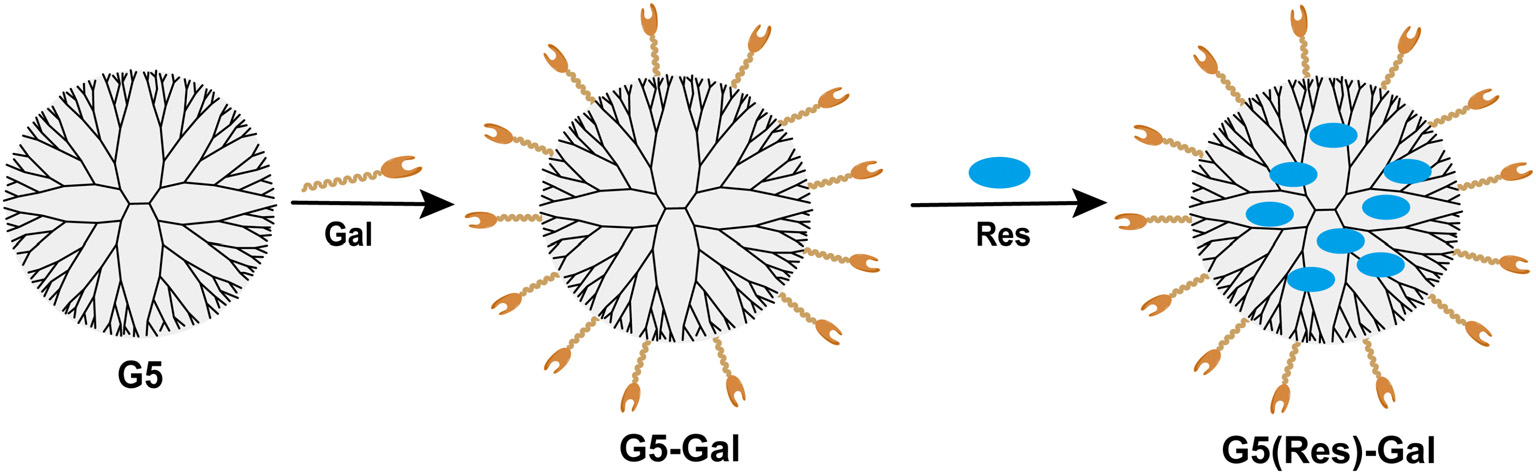
The design of G5-Gal nanocarriers and G5(Res)-Gal nanomaterials.

Herein, we designed a glycosylated dendrimer-based nanomedicine (**Figure 1**) and studied whether the fifth-generation (G5) PAMAM dendrimer modified by galactose (G5-Gal) has the effect of targeting hepatocellular carcinoma cells. Then, after synthesizing the Res-loaded glycosylated dendrimers (G5(Res)-Gal), we explore the effect of G5(Res)-Gal on hepatocellular carcinoma cells *in vitro*. In this study, the synthetized G5-Gal were analyzed by ^1^H NMR spectroscopy and zeta nanoparticle potentiometer analysis to disclose the physical property in detail. The antifouling property of the formed G5-Gal was tested *via* protein resistance assay, and the targeting capability of G5-Gal was measured by flow cytometry. Furthermore, the G5(Res)-Gal complexes were characterized using UV spectrophotometry and the impact of G5(Res)-Gal on normal liver cells as well as its cytotoxicity against different types of hepatocellular carcinoma cells was evaluated by CCK-8 assay. Finally, cell apoptosis experiments were conducted to further evaluate the effects of G5(Res)-Gal on Hepa1-6 cells. These results are essential for clear interpretation of G5(Res)-Gal for their further design and biomedical uses.

## 2 Materials and methods

### 2.1 Synthesis and characterization of G5-Gal nanocarriers

The 1-ethyl-3-(3-dimethylaminopropyl) carbodiimide (EDC) coupling strategy was applied to form G5-Gal nanocarriers. In brief, a dimethyl sulfoxide (DMSO) solution of EDC (99.76 mg, 5 mL) was dropwise added to a DMSO solution containing Gal (11.04 mg), and the mixture was stirred for 30 min at room temperature. Then, the mixture was added with N-hydroxysuccinimide (NHS) (59.89 mg) dissolved in DMSO (5 mL) and continued stirring for 3 h. Thereafter, G5 dendrimers (50 mg) dissolved in DMSO (5 mL) were added to the above mixture while stirring at room temperature for another 3 days. Finally, the mixture was collected and dialyzed against water (nine times, 2 L) for 3 days using a dialysis membrane with an MWCO of 1,000. After a further lyophilization process, G5-Gal nanocarriers were obtained and stored at −20°C for further use.

A Bruker AV-600 NMR spectrometer was used to measure ^1^H nuclear magnetic resonance (NMR) for characterizing the prepared G5-Gal nanocarriers. G5 dendrimers and G5-Gal nanocarriers were dissolved and dispersed in deuterium oxide (D_2_O) before measurements. Then, the proportion of Gal modified on G5 is calculated by the NMR integration of characteristic peak. Subsequently, Fourier transform infrared (FTIR) spectra were recorded on a Nicolet 6700 FTIR spectrophotometer (Thermo Electron Corporation, Madison, WI). G5 dendrimers or G5-Gal nanocarriers were mixed with milled KBr crystals and pressed to form 13-mm-diameter disks before measurements.

Zeta potential and dynamic light scattering (DLS) measurements were performed using a Malvern Zetasizer Nano ZS model ZEN3600 (Worcestershire, UK) coupled with a standard 633-nm laser. All samples were dispersed in water before measurements. Meanwhile, the colloidal stability of the G5-Gal nanocarriers was inspected by dispersing them in water (1 mg/mL) and monitoring their hydrodynamic size changes within 7 days at room temperature by DLS.

The protein resistance property of G5-Gal nanocarriers was evaluated by co-incubating bovine serum albumin (BSA) with G5-Gal nanocarriers. In brief, the G5-Gal nanocarriers at different concentrations (0.5 mg/mL, 1 mg/mL, and 2 mg/mL, respectively, in water) were incubated with BSA (2 mg/mL, in water) at 37°C for 4 h. Each mixture was centrifuged (8,000 rpm/min, 5 min), and the supernatant was detected using a Lambda 25 UV-vis spectrophotometer (Perkin Elmer, Waltham, MA) according to the standard instruction of the BCA quantitation kit. The difference in the concentration of BSA in the supernatant after G5-Gal incubation and that before G5-Gal incubation was used as an indicator to characterize the antifouling property of each sample. Moreover, G5 dendrimers incubated with BSA were used as control.

### 2.2 Exploration on targeting property of G5-Gal nanocarriers

First of all, the G5-Gal were reacted with 10 molar equivalents of Cyanine5 NHS (Cy5-NHS) DMSO under vigorous magnetic stirring for 24 h to get the raw product of Cy5-G5-Gal. The raw product was collected and dialyzed against water (nine times, 2 L) for 3 days using a dialysis membrane with an MWCO of 3,500. Then, the Cy5-G5-Gal were obtained after a further lyophilization process. Subsequently, the UV-vis spectrophotometer was used to determine the average number of Cy5 moieties attached on the surface of each G5-Gal. Meanwhile, Cy5-G5 complexes were also formed and characterized for comparison.

Next, Hepa1-6 cells (a mouse hepatocellular carcinoma cell line) were regularly cultured and passaged with DMEM supplemented with 10% FBS, 1 mM sodium pyruvate, 100 U/mL penicillin, and 100 U/mL streptomycin (named DMEM^+++^) at 37°C and 5% CO_2_. The cellular uptake assay was performed to evaluate the targeting property of G5-Gal nanocarriers. In brief, Hepa1-6 cells were seeded into a 12-well plate at a density of 1.0 × 10^5^ cells per well and were cultured overnight at 37°C and 5% CO_2_. Then, the cell medium of each well was replaced with 500 μL DMEM^+++^ containing Cy5-G5-Gal or Cy5-G5 polyplexes (2 μM per well). After the cells were incubated for 4 h, the cells were washed with PBS for three times, digested with trypsin, collected into the tube, resuspended in PBS after centrifugation, and finally measured in the FL2-H channel by flow cytometry.

### 2.3 Synthesis and characterization of G5(Res)-Gal nanomedicine

Res was physically encapsulated within G5-Gal. The molar feeding ratio of G5-Gal and Res was set at 1: 20. In brief, the Res (2.8 mg) in 500-μL methanolic solution was dropped to an aqueous solution of G5-Gal (20 mg, 5 mL) under stirring in the dark overnight. After evaporation of the methanol solvent, the mixture solution was centrifuged (8,000 r/min, 10 min) to get the G5(Res)-Gal nanomedicines in the supernatant. Meanwhile, the precipitate associated with free Res was redissolved in methanol and quantified using UV-vis spectrophotometry according to standard Res absorbance at the 306-nm/concentration curve measured by a Lambda 25 UV-vis spectrophotometer. The Res loading content (LC) and encapsulation efficiency (EE) were calculated according to the following equations: LC (%) = (the mass of loaded Res within the complexes/the mass of Res-loaded complexes) × 100% (i); and EE (%) = (the mass of loaded Res within the complexes/the initial mass of Res) × 100% (ii).

The formed Res complexes were characterized with UV-vis spectroscopy. In addition, *in vitro* Res’s release kinetics of G5(Res)-Gal nanomedicines was also tested by UV-vis spectroscopy under two different pHs (pH = 7.4 and pH = 5.4) at 37°C. The G5(Res)-Gal nanomedicines (2 mg) were dispersed in 1-mL phosphate buffer at pH 5.5 or pH 7.4, transferred to a dialysis bag (MWCO = 3500 Da), and submerged in the corresponding buffer medium (9 mL). It is worth noting that 1% DMSO should be added to phosphate buffer to help dissolve the released free Res. The whole system was kept in a constant temperature vibration shaker at 37°C for different time periods. At each scheduled time interval, 1 mL of the external buffer medium was collected and measured by UV-vis spectroscopy, and then the same volume of the corresponding buffer medium was replenished. The experiment was performed in triplicate for each sample.

### 2.4 Effects of G5(Res)-Gal nanomedicine on normal hepatic cells and hepatocellular carcinoma cells

AML-12 cells as normal hepatic cells and Hepa1-6 cells as hepatocellular carcinoma cells were selected to evaluate the effect of G5(Res)-Gal nanomedicine on cell viability by Cell Counting Kit-8 (CCK-8) assay. Taking Hepa1-6 cells as an example, Hepa1-6 cells were first seeded in 96-well plates at a density of 5 × 10^3^ cells per well with 100 μL DMEM^+++^ and incubated overnight. The next day, the medium in each well was replaced with fresh DMEM^+++^ containing G5(Res)-Gal at various Res concentrations (50 μM, 100 μM, 200 μM, 300 μM, 400 μM, and 500 μM, respectively). After 24 h, each well was added with 10-μL CCK-8 solution and the cells were cultured for additional 2 h. The assay was carried out according to the manufacturer’s instruction, and the absorbance of each well was recorded by a Thermo Scientific Multiskan MK3 ELISA reader (Waltham, MA) at 450 nm. Hepa1-6 cells treated with PBS or G5-Gal were used as control, the half-maximal inhibitory concentrations (IC_50_s) of G5(Res)-Gal nanomedicines were calculated using a GraphPad Prism software (GraphPad Software Inc., San Diego, CA). Each sample was tested in sextuplicate wells. It is worth noting that the chemotherapy effect of G5(Res)-Gal nanomedicines on other hepatocellular carcinoma cells (SNU398 cells and HepG2 cells) was also evaluated by CCK-8 assay, and the steps were the same as described above.

In addition, the apoptosis of Hepa1-6 cells induced by G5(Res)-Gal nanomedicines was examined using an Annexin V-FITC/PI apoptosis detection kit. In brief, Hepa1-6 cells were cultured in six-well plates at a density of 2 × 10^5^ cells per well in 2 mL of DMEM^+++^, incubated with G5(Res)-Gal nanomedicines at a Res concentration of 200 μM for 24 h. Subsequently, the cells in each well were washed, collected, resuspended in 195 μL of binding buffer, and added with 5 μL of Annexin V-FITC and 10 μL of PI according to the kit’s instruction. Then, each sample was incubated at 0°C for 15 min in the dark before flow cytometry analysis. Cells treated with PBS were used as control. For each sample, 2 × 10^4^ cells were counted, and each measurement was repeated for three times.

### 2.5 Statistical analysis

All data were shown as mean ± standard deviation (SD, n ≥ 3). The significant difference of experimental results between groups was analyzed by one-way analysis of variance statistical method using the IBM SPSS Statistic 26 software (IBM, Armonk, NY). The significance level (*p*) was set at 0.05, 0.01, or 0.001, respectively.

## 3 Results and discussion

### 3.1 Synthesis and characterization of G5-Gal nanocarriers

**Figure 2:**
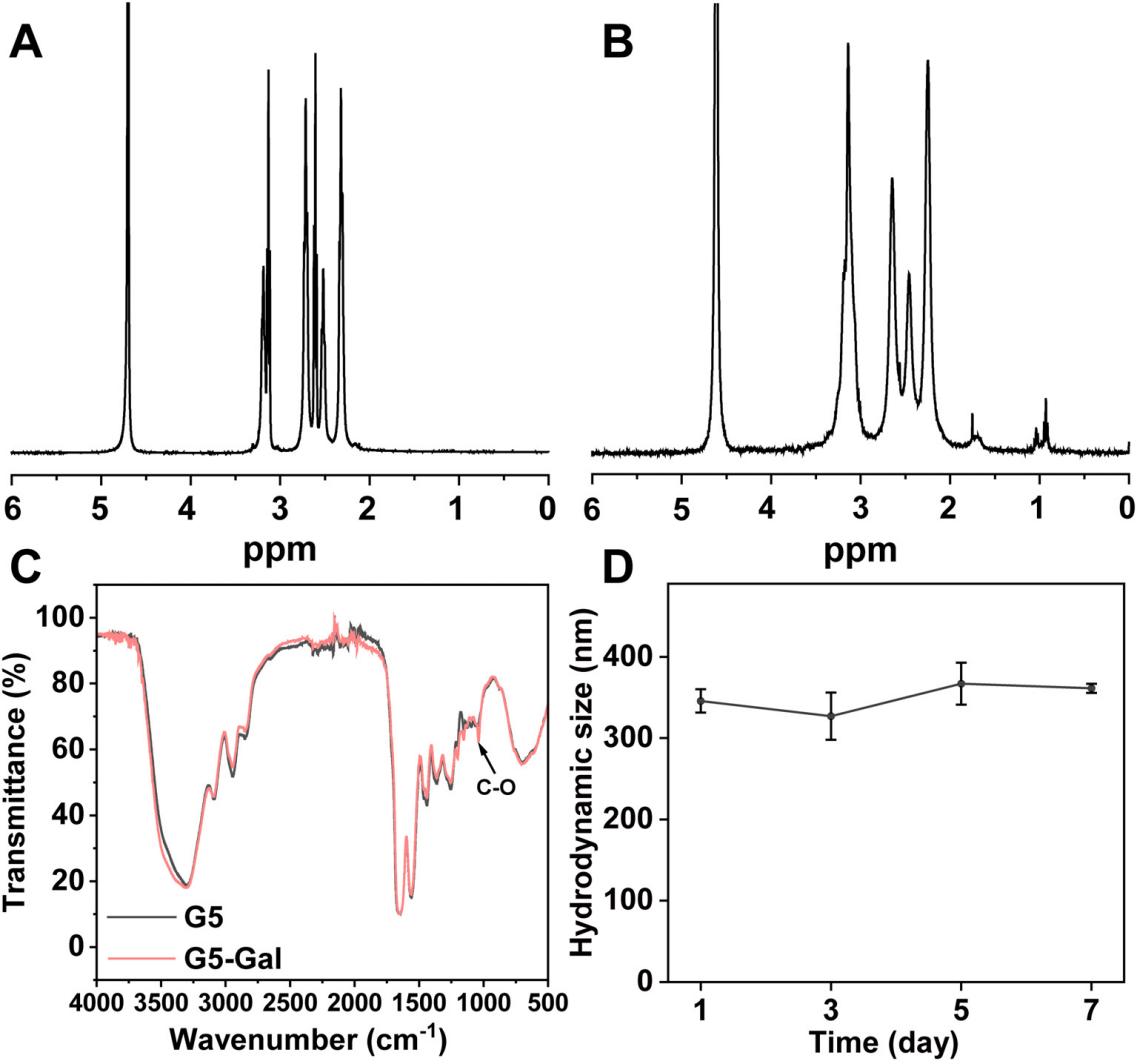
Characterization of G5-Gal nanocarriers. ^1^H NMR spectra of G5 **(A)** and G5-Gal **(B)**. **(C)** FTIR spectra of G5 and G5-Gal. **(D)** Hydrodynamic size change of G5-Gal (1 mg/mL) dispersed in water within 7 days.

The EDC coupling strategy utilized for the formation of G5-Gal nanocarriers is illustrated in **Figure 1**. Moreover, both the G5 dendrimers and the synthesized G5-Gal nanocarriers were characterized using ^1^H NMR. As illustrated in **Figures 2A, B**, based on the NMR integration of the characteristic peak at 2.2 ppm–3.4 ppm for the G5 dendrimer and at 3.4 ppm–4.5 ppm for Gal, it can be inferred that each G5 dendrimer was conjugated with approximately 15 Gal molecules. Subsequently, the G5 dendrimers and G5-Gal nanocarriers were analyzed using FTIR spectra. As shown in **Figure 2C**, the peak observed at 1,100 cm^−1^ corresponds to the C–O vibration signal in G5-Gal, indicating the successful synthesis of G5-Gal nanocarriers.

Next, the hydrodynamic sizes, polymer dispersity index (PDI) and zeta potentials of G5 dendrimers and G5-Gal nanocarriers were investigated (**Supplementary Table S1**). After functional modification of G5, the hydrodynamic size and zeta potential of G5-Gal are 345.83 ± 14.28 nm (PDI: 0.26 ± 0.02) and 41.91 ± 0.64 mV, respectively. Subsequently, the colloidal stability of G5-Gal was assessed. After the formed G5-Gal nanocarriers were dispersed into water, stored for 7 days at room temperature, there is no precipitation occurred during the storage time period, and the hydrodynamic sizes of G5-Gal nanocarriers are fluctuated around 327.1 nm–367.0 nm (**Figure 2D**), suggesting the stability of the G5-Gal.

**Figure 3:**
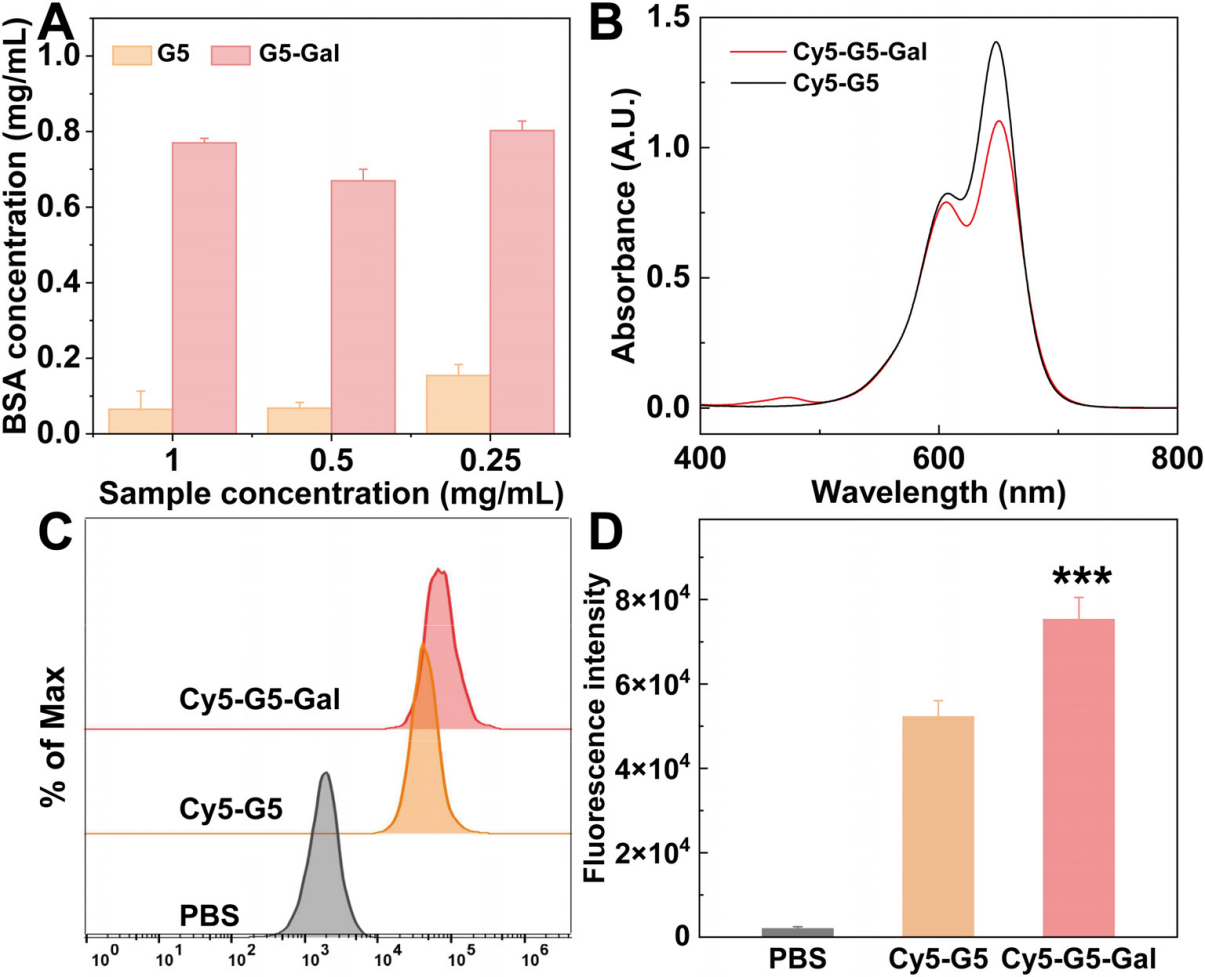
**(A)** UV-vis spectral quantification of the BSA concentration in the supernatant of G5/BSA or G5-Gal/BSA after centrifugation. **(B)** UV-vis spectra of Cy5-G5 and Cy5-G5-Gal. **(C, D)** Flow cytometry assay of Hepa1-6 cells treated with PBS, Cy5-G5, and Cy5-G5-Gal for 4 (h). In **(D)** *** for p < 0.001.

In addition, the antifouling property of nanomaterials is essential for their application in tumor delivery. Therefore, a protein resistance assay was conducted to evaluate the antifouling capability of the formed G5-Gal. The BSA concentration in the mixture solution of G5-Gal/BSA before and after centrifugation was measured through UV-vis spectral analysis. The higher BSA absorbance means the better the protein resistance of the nanomaterials. As shown in **Figure 3A**, the BSA absorbance after its interaction with G5-Gal or G5 under the same G5 concentration was recorded. Under the same G5 concentration, the BSA concentration for the G5 group is significantly less than that for the G5-Gal group. This suggests that due to the surface Gal modifications, G5-Gal can be rendered with good antifouling property.

### 3.2 Targeting properties of G5-Gal nanocarriers

The binding of galactose to specific receptors on the surface of hepatocellular carcinoma, such as the asialoglycoprotein receptor (ASGPR), has been reported to trigger receptor-mediated endocytosis (26). This pattern of phagocytosis typically exhibits high specificity and efficiency. Therefore, prior to estimation of the drug loading capacity of G5-Gal nanocarriers, the ability of G5-Gal nanocarriers to target hepatocellular carcinoma cells was first tested using Hepa1-6 cells *in vitro*. First, Cy5-labeled G5-Gal (Cy5-G5-Gal) and G5 (Cy5-G5) were synthesized separately to facilitate tracking in cells. The UV-vis spectrum shows the successful modification of Cy5 (Cy5 characteristic peak at 646 nm) (**Figure 3B**). After calculation, the number of Cy5 onto each G5-Gal or G5 is 4.5 and 5.4, respectively. Thereafter, Cy5-G5-Gal and Cy5-G5 were incubated with Hepa1-6 cells for 4 h. Flow cytometry were finally used to observe and quantify the fluorescence changes of the Hepa1-6 cells. As shown in **Figures 3C, D**, both Cy5-G5-Gal and Cy5-G5 can be internalized by cells. Furthermore, the Hepa1-6 cells treated with Cy5-G5-Gal showed higher fluorescence intensity than that of Cy5-G5, although the fluorescence intensity of Cy5-G5-Gal was lower than that of Cy5-G5 under the same G5 condition. We believe that this disparity arises from non-specific interaction-mediated cellular uptake of Cy5-G5, resulting in low phagocytic efficiency, whereas specific binding facilitates high phagocytic efficiency for the entry of Cy5-G5-Gal into cells. This unequivocally demonstrates the targeted ability of Gal toward Hepa1-6 cells.

### 3.3 Characterization of G5(Res)-Gal nanomedicine

**Figure 4:**
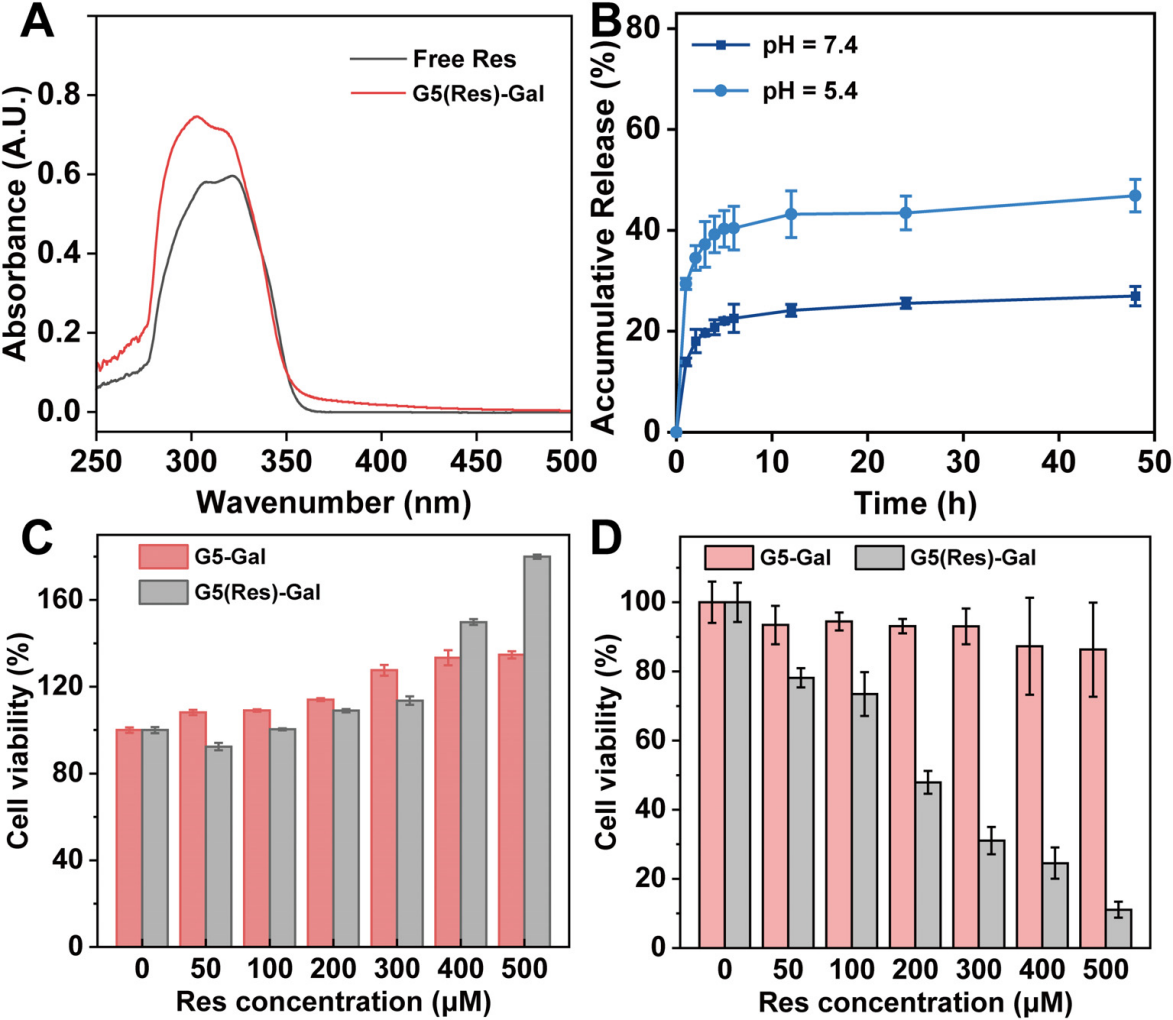
**(A)** UV-vis absorption spectra of free Res and G5(Res)-Gal complexes. **(B)** Res release profiles from G5-Gal in PBS at pH = 5.5 and pH = 7.4 at 37°C, respectively. The cytotoxicity of G5-Gal and G5(Res)-Gal to AML12 cells **(C)** or Hepa1-6 cells **(D)** for 24 h at various concentrations of Res. G5-Gal had an equivalent molar concentration to the G5(Res)-Gal.

Encouraged by the above experimental results, G5-Gal were further used as nanocarriers for physical encapsulation with Res (G5(Res)-Gal), which was identified through UV-vis spectrometry. By calculation from standard curve, the encapsulation efficiency (EE) of Res within the G5-Gal is 77.3% (**Supplementary Table S2**), corresponding to each G5-Gal complexed with 10 Res molecules. The UV-vis spectra of G5(Res)-Gal and free Res are shown in **Figure 4A**. Clearly, G5(Res)-Gal show the featured absorption peak at 306 nm, which should be attributed to the loaded Res. Next, the release kinetics of Res from the G5(Res)-Gal nanomedicine was examined under different pHs (pH 5.4 and pH 7.4) *in vitro* (**Figure 4B**). The release rate of Res from the G5(Res)-Gal nanomedicine at pH 5.4 is much greater than at pH 7.4. Less than 25% of Res is released from the G5(Res)-Gal nanomedicine under pH 7.4 within 48 h, whereas approximately 43% of Res is released at pH 5.4 under similar time points. The faster release of Res under the slight acidic pH conditions should be attributed to the increased concentration of hydrogen ions and hydroxyl ions in the solution at low pH, leading to alterations in the hydrophobic environment surrounding Res and dendrimers. This alteration may weaken the hydrophobic interaction between them, facilitating an easier release of Res. This phenomenon would be beneficial for the treatment of tumors with the slightly acidic tumor microenvironment.

### 3.4 Effects of G5(Res)-Gal nanomedicine on normal hepatic cells and hepatocellular carcinoma cells

We selected AML-12 cells, Hepa1-6 cells, SNU398 cells, and HepG2 cells to evaluate the effect of the G5(Res)-Gal nanomedicine on cell activity by CCK-8 assay. As shown in **Figure 4C**, the viability of AML-12 cells incubated with either G5(Res)-Gal or G5-Gal remains consistently above 90%, irrespective of the concentration of Res, which indicates that both G5-Gal nanocarriers and G5(Res)-Gal nanomedicine are non-toxic to normal hepatic cells. Similarly, the viability of Hepa1-6 cells after treated with G5(Res)-Gal or G5-Gal was also evaluated *via* CCK-8 assay (**Figure 4D**). Obviously, the drug-free G5-Gal display excellent cytocompatibility with cell viability reaching above 86% under all the equivalent Res concentrations studied. To be opposed, the G5(Res)-Gal has significant inhibitory effects on Hepa1-6 cells. In addition, the inhibitory effects of the G5(Res)-Gal nanomedicine on SNU398 cells or HepG2 cells were also dose-dependent (**Supplementary Figure S1**). Through calculation, the IC_50_ values of G5(Res)-Gal for Hepa1-6 cells, SNU398 cells, and HepG2 cells is 174.2 μM, 167.7 μM, and 188.2 μM, respectively, suggesting that G5(Res)-Gal can inhibit the growth of hepatocellular carcinoma cells within certain dose range of Res.

**Figure 5:**
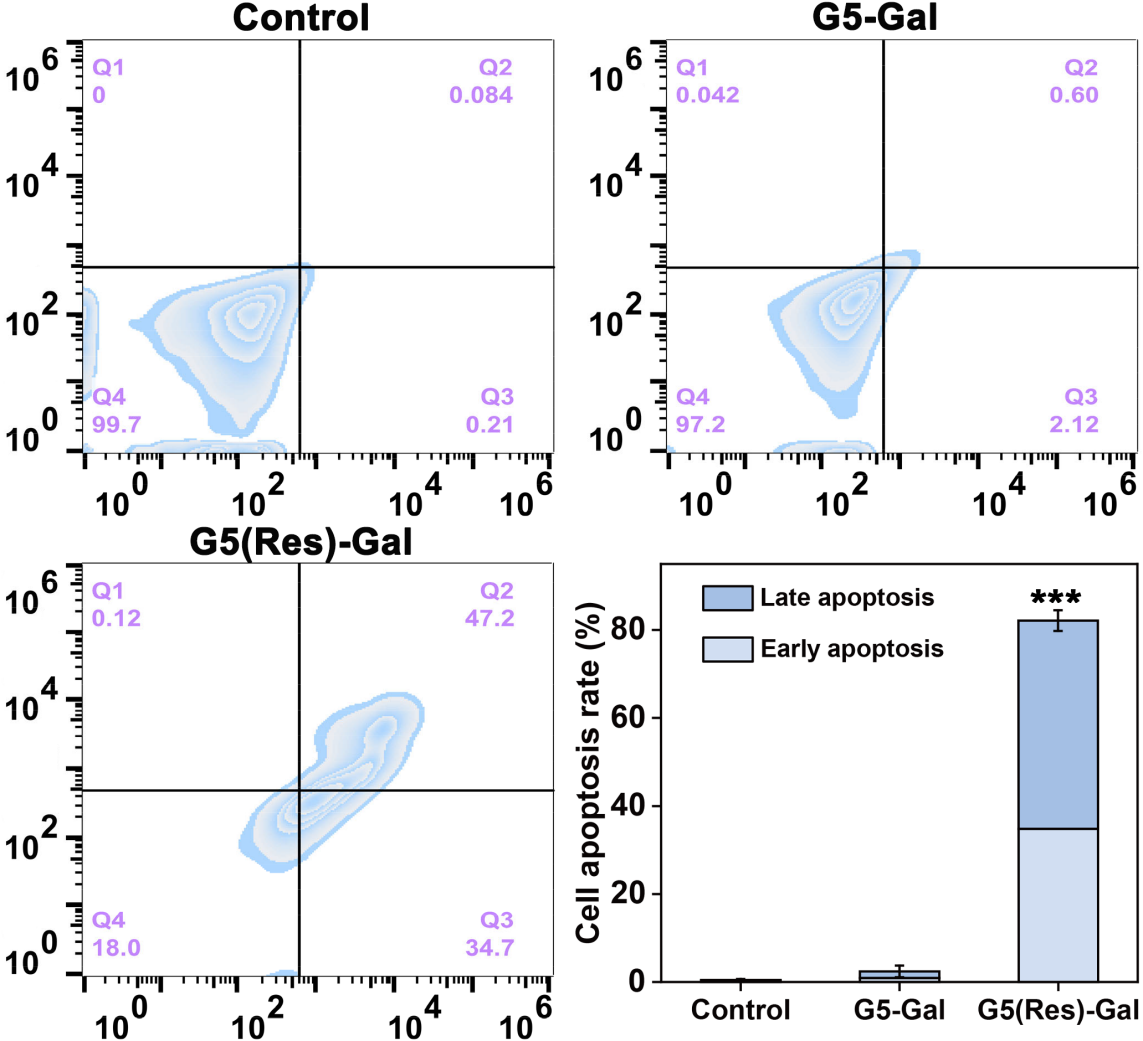
Flow cytometry and quantitative analysis of Hepa1-6 cell apoptosis after incubation with PBS, G5-Gal, and G5(Res)-Gal for 24 h (mean ± SD, n = 3). In the last picture, *** for p < 0.001.

Furthermore, cell apoptosis assays were selected to reveal the apoptosis efficiency of Hepa1-6 cells after treatment with the G5(Res)-Gal nanomedicine for 24 h. The representative density plots are shown in **Figure 5**. The G5(Res)-Gal nanomedicine markedly promote cell apoptosis as compared with the Hepa1-6 cells incubated with both PBS and G5-Gal.

## 4 Conclusion

In summary, this study aimed to design and synthesize a glycosylated dendrimer using EDC coupling chemistry. Moreover, Res was physically encapsulated inside the glycosylated dendrimer, resulting in the formation of a hepatocellular carcinoma cell-targeting drug system. The synthesized G5-Gal exhibited favorable colloidal stability and anti-protein adsorption properties, enabling specific targeting toward hepatocellular carcinoma cells. Moreover, the formed G5(Res)-Gal significantly enhanced the water solubility of Res and demonstrated remarkable *in vitro* chemotherapy effects against hepatocellular carcinoma cells at non-toxic doses to normal liver cells. This study provides a theoretical basis and technical support for enhancing the application of resveratrol and developing its delivery system. Additionally, it offers important insights into targeted nanomedicine development and therapeutic strategies for hepatocellular carcinoma.

## Data Availability

The datasets presented in this study can be found in online repositories. The names of the repository/repositories and accession number(s) can be found in the article/**Supplementary Material**.
