# Supplementary material for: Effects of resveratrol-loaded dendrimer nanomedicine on hepatocellular carcinoma cells
Source: Front Immunol. 2024 Nov 14;15:1500998. doi: 10.3389/fimmu.2024.1500998 (PMC11602518; doi:10.3389/fimmu.2024.1500998)
Supplement: Supplementary file 1 [file DataSheet1.docx]

Supplementary Material

Effects of resveratrol-loaded dendrimer nanomedicine on hepatocellular carcinoma cells

Jiao Qu^1^, Yueqin Zhang^2^, Cong Song^2^, and Yue Wang^1*^

^1^Department of Radiology, Songjiang Hospital Affiliated to Shanghai Jiao Tong University School of Medicine, Shanghai 201600, China

^2^Medical Science and Technology Innovation Center, Shandong First Medical University, Jinan, Shandong 250117, China

*Corresponding authors: Yue Wang: wymyf@163.com

**Part of experimental details:**

**Materials.** Ethylenediamine core amine-terminated generation 5 (G5) poly(amidoamine) (PAMAM) dendrimers was purchased from Dendritech (Midland, MI). Acetic anhydride, triethylamine, N-hydroxysuccinimide (NHS), and 1-ethyl-3-(3-dimethylaminopropyl) carbodiimide hydrochloride (EDC.HCl) were obtained from Sinopharm Chemical Reagent Co., Ltd. (Shanghai, China). Dimethyl sulfoxide (DMSO) was acquired from Shanghai Lingfeng Chemical Reagent Co., Ltd. (Shanghai, China). Galactose and regenerated cellulose dialysis membranes with a molecular weight cut-off (MWCO) of 1000 was acquired from Shanghai Yuanye Biotechnology Co, Ltd. (Shanghai, China). AML-12 cells (a normal hepatic cell line), SNU398 cells (a hepatocellular carcinoma cell line), HepG2 cells (a hepatocellular carcinoma cell line) and Hepa1-6 cells (a hepatocellular carcinoma cell line) were from Institute of Biochemistry and Cell Biology (the Chinese Academy of Sciences, Shanghai, China). Dulbecco’s Modified Eagle Medium (DMEM), fetal bovine serum (FBS), 0.25% trypsin-ethylene diamine tetraacetic acid (EDTA), penicillin and streptomycin were from Gibco (Carlsbad, CA). Cell Counting Kit-8 (CCK-8) was from Beyotime Biotech Co, Ltd. (Shanghai, China). Phosphate buffered saline (PBS) and annexin V-FITC/PI (fluorescein isothiocyanate/propidium iodide) apoptosis detection kit were from Wuhan Servicebio Biological Technology Co., Ltd. (Wuhan, China). Water used in all experiments was purified using a PURIST UV Ultrapure Water System (RephiLe Bioscience, Ltd., Shanghai, China) with a resistivity higher than 18.2 MΩ·cm.

**Table S1.** Hydrodynamic sizes and zeta potentials of G5 and G5-Gal dendrimers dispersed in water

| Sample | Hydrodynamic size (nm) | Polydispersity index (PDI) | Zeta potential (mV) |
| --- | --- | --- | --- |
| G5 | 258.43 ± 13.23 | 0.38 ± 0.02 | 0.38 ± 0.02 |
| G5-Gal | 345.83 ± 14.28 | 0.26 ± 0.02 | 0.38 ± 0.02 |

**Table S2.** The encapsulation efficiency (EE) and Loading content (LC) of Res-based nanocomplexes

| Sample | Encapsulation efficiency (EE, %) | Loading content (LC, %) |
| --- | --- | --- |
| G5(Res)-Gal | 6.6% | 77.3% |

## Supplementary Figures


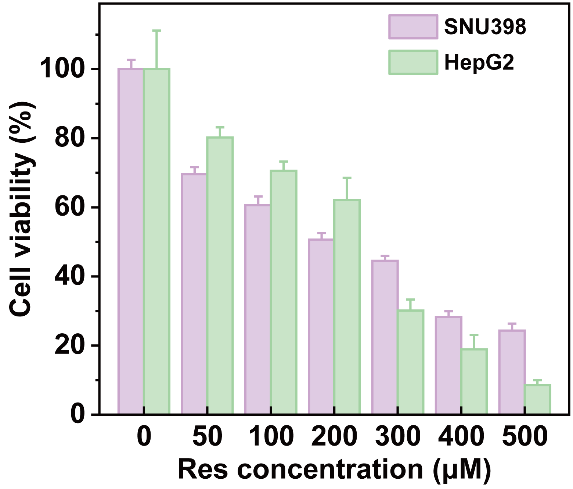


**Supplementary Figure 1.** The cytotoxicity of G5(Res)-Gal to HepG2 cells or SNU398 cells for 24 h at various concentrations of Res.
